# Treatment of Nodular Basal Cell Carcinoma With Topical 5% Imiquimod Cream: An Autobiographical Case Report

**DOI:** 10.7759/cureus.108890

**Published:** 2026-05-15

**Authors:** Philip R Cohen

**Affiliations:** 1 Dermatology, University of California, Davis Medical Center, Sacramento, USA

**Keywords:** basal, cancer, carcinoma, cell, imiquimod, infection, micrographic, mohs, skin, surgery

## Abstract

The most common skin cancer is basal cell carcinoma. The neoplasm is characterized by several different morphologic presentations; the most frequent morphologic variants are superficial and nodular. Each clinical presentation of the tumor has a corresponding histologic presentation. The biological behavior of a basal cell carcinoma depends not only on its histologic subtype but also on its location. Surgical excision is the treatment of choice for most basal cell carcinomas; this intervention is typically used for basal cell carcinomas that demonstrate aggressive behavior. Nonsurgical treatment, such as topical 5% imiquimod cream, may be considered for lower-risk tumors. After consultation with the dermatologist, I (a 65-year-old man) decided to treat my primary nodular basal cell carcinoma on my right periocular temple by applying 5% imiquimod cream daily to the neoplasm and the adjacent skin. The cancer was treated for a total of 45 days; during treatment, I had to discontinue the therapy twice because I developed severe, medication-related, local cutaneous adverse side effects, which included inflammation, eschar, and ulcers. I achieved complete clinical clearance of the carcinoma; the only residual finding at the previous tumor site was an atrophic patch. The management of basal cell carcinoma includes noninvasive and invasive modalities. Mohs micrographic surgery is the treatment of choice for most facial basal cell carcinomas and tumors of infiltrative histologic subtype. Mohs surgery is an invasive procedure; it can be associated with not only postoperative complications and scar but also possibly inferior cosmetic results. However, the advantages of Mohs surgery are not only its 97.5% cure rate for primary basal cell carcinomas but also the very low rate of tumor persistence following the procedure. Superficial basal cell carcinomas on the neck, trunk, or extremities can be treated with a daily topical application of 5% imiquimod cream, five times per week, for six weeks. In contrast to surgical excision, topical imiquimod has a lower cure rate; however, it is a noninvasive therapy and may result in cosmetically superior results. Nodular basal cell carcinomas, including periocular tumors, have also been successfully treated with topical 5% imiquimod cream. Treatment using topical 5% imiquimod cream for a basal cell carcinoma, compared to Mohs surgical excision, has several potential disadvantages. Some of these include a lower cure rate, a longer treatment duration (such as six weeks with five daily applications each week to treat a superficial basal cell carcinoma versus a single day of surgery), and the development of localized adverse cutaneous reactions that occur both on the tumor-containing epithelium and the skin surrounding the cancer. In conclusion, the management of basal cell carcinoma is an individualized decision for which several factors need to be evaluated. Although surgery is the gold standard of treatment, patients who do not want surgical intervention and possibly a better cosmetic result without a surgical scar may appropriately elect potentially effective noninvasive therapies, which have lower cure rates, longer treatment duration, and more frequently have associated adverse cutaneous reactions. This experience has taught me as a patient and as a physician that the topical management of a basal cell carcinoma with 5% imiquimod cream can result in more complicated adverse cutaneous effects than anticipated.

## Introduction

Basal cell carcinoma is the most common cancer of the skin. There are several clinical presentations of the neoplasm, and each has corresponding pathologic features. The aggressiveness of the tumor is predominantly based on the location and the microscopic changes demonstrated by the tumor [1-4].

Treatment of basal cell carcinoma is based on the biological behavior of the tumor [1-4]. Excision (Mohs or surgical removal without margin control at the time of tumor extirpation) of the cancer is the standard therapy for the management of a basal cell carcinoma [5-7]. For a lower-risk tumor, a nonsurgical treatment, such as topical 5% imiquimod cream, may be considered [8-10].

I, a 65-year-old man, presented with a biopsy-confirmed nodular basal cell carcinoma on my right periocular temple. I elected a nonsurgical treatment consisting of a daily topical application of 5% imiquimod cream for 45 days instead of an excision using the Mohs micrographic surgical technique. I needed to interrupt treatment twice to allow the severe local cutaneous reaction to the medication to heal before resuming therapy; my tumor completely clinically resolved after treatment with 5% imiquimod cream. This experience has taught me as a patient and as a physician that the topical management of a basal cell carcinoma with 5% imiquimod cream can result in more complicated adverse cutaneous effects than anticipated.

## Case presentation

**Figure 1:**
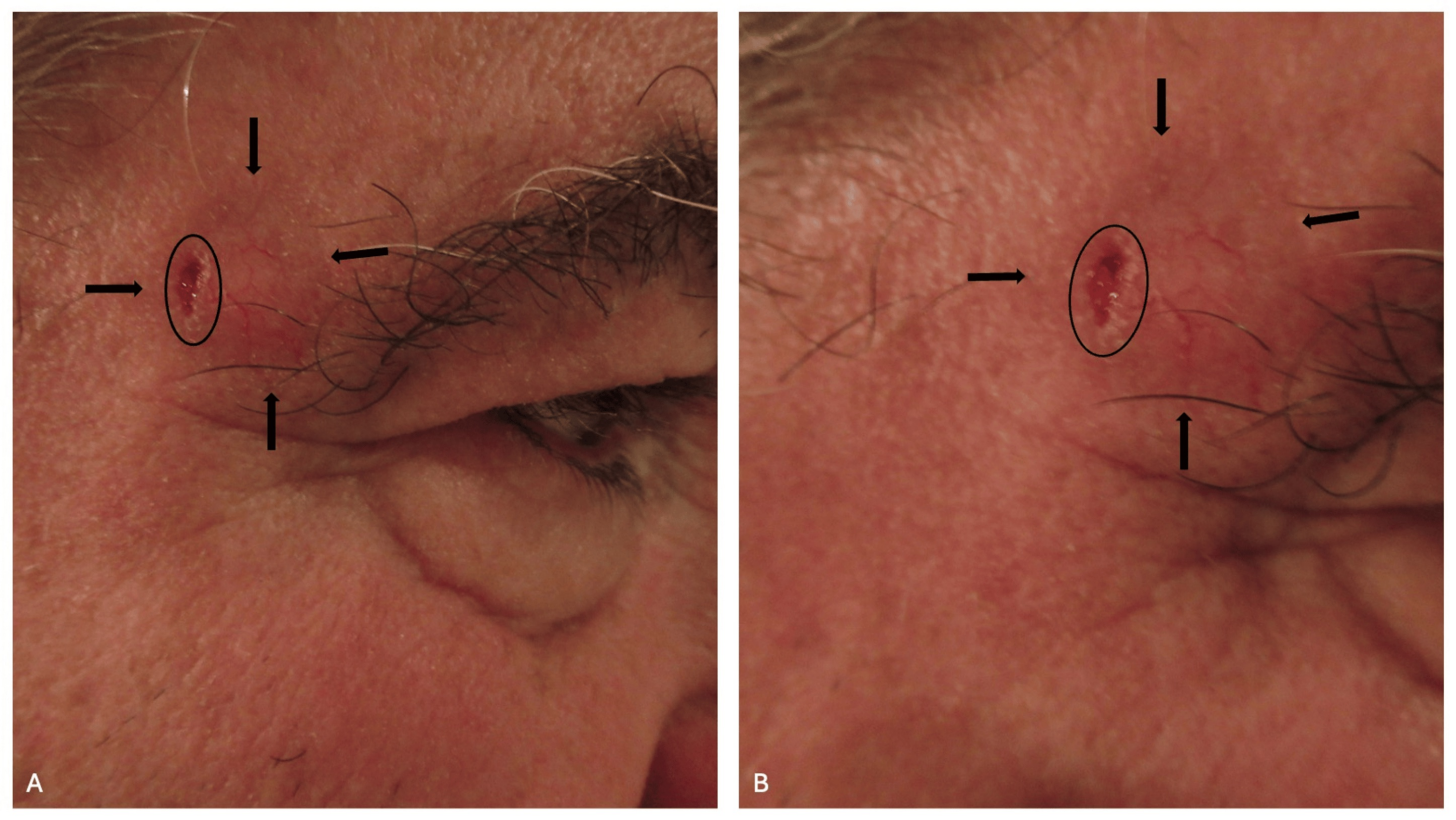
Clinical presentation of a nodular basal cell carcinoma on the right temple Distant (A) and closer (B) views of a nodule with prominent telangiectasias. The clinical margins of the tumor are demonstrated by the black arrow. A spontaneous ulcer in the lateral portion of the tumor (within the black oval) developed in the lateral portion of the neoplasm. I consent to the publication of the images and my eyes not being blocked.

I, a 65-year-old Caucasian man, presented with an asymptomatic red nodule on my right temple that had been slowly and progressively increasing in size for approximately nine months; I had no previous history of skin cancer. Initially, the lesion was entirely red, and I thought it was folliculitis. The papule grew in diameter, and I considered the possibilities of a mosquito bite; when the lesion remained and became a nodule, I entertained the possibility of a persistent insect bite reaction. Eventually, an ulcer appeared, and small dilated vessels were noted; I finally suspected that it was a basal cell carcinoma (Figure 1).

My past medical history was significant for an ischemic stroke (secondary to long-distance running-associated atrial flutter), hyperlipidemia, hypertension, gout, severe bilateral spinal stenosis involving multiple lumbar vertebrae, and bilateral extracapsular cataract extraction and intraocular lens placement. My current daily medications include apixaban, ezetimibe, magnesium glycinate, metoprolol, valsartan, and vitamin C.

**Figure 2:**
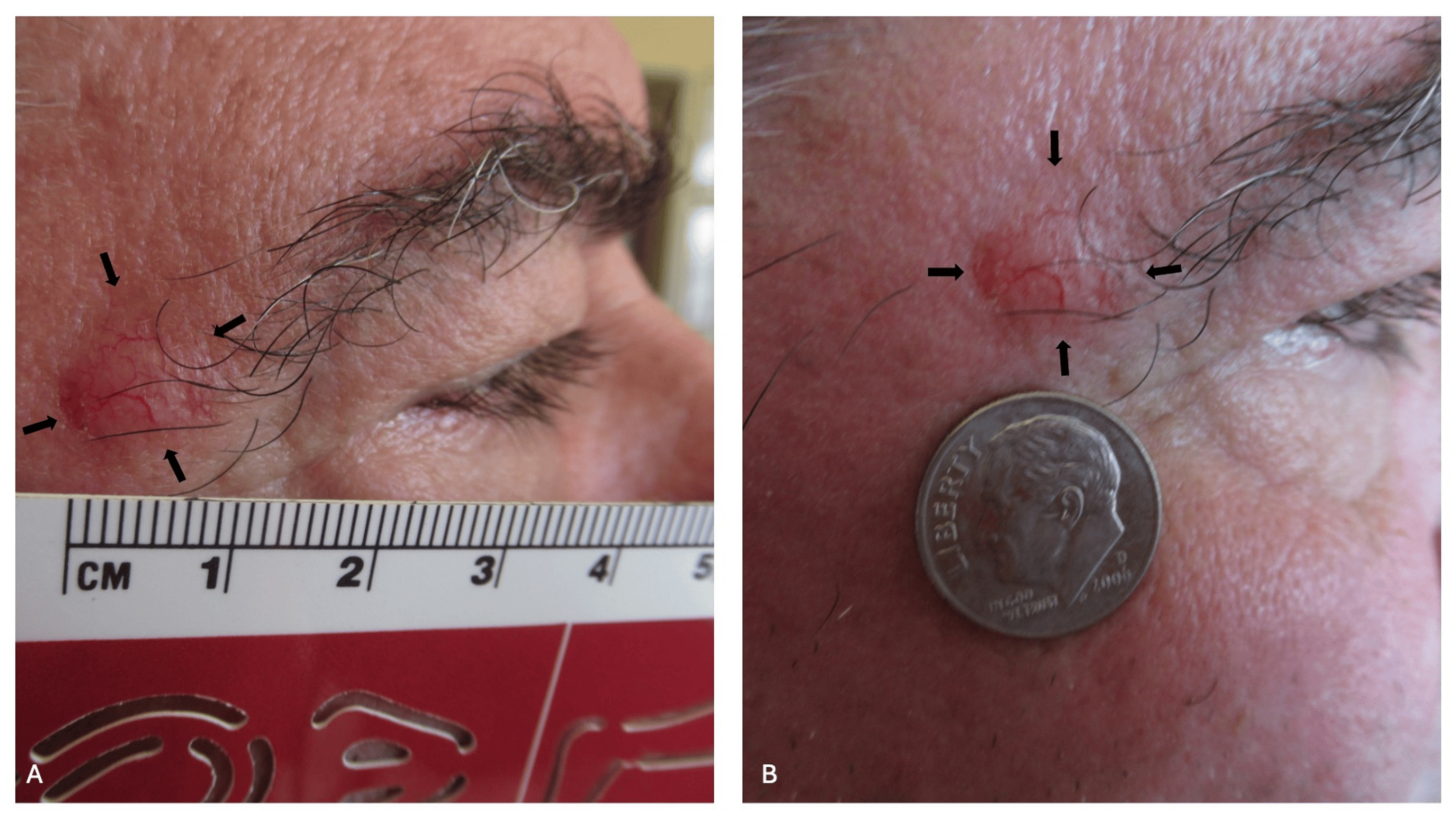
Morphology of a periocular nodular basal cell carcinoma The borders of the basal cell carcinoma are demonstrated using black arrows. The previous ulcer had nearly healed spontaneously (A and B). A ruler was used to measure the 9 x 9 mm size of the tumor (A). The tumor was approximately the size of a dime (B). I consent to the publication of the images and my eyes not being blocked.

Clinical examination of my right temple showed a 9 x 9 mm telangiectatic periocular nodule (Figure 2). A shave biopsy was performed. Microscopic examination demonstrated nodular aggregates of basaloid tumor cells in the dermis. Correlation of the clinical morphology and the pathologic changes established the diagnosis of a nodular basal cell carcinoma.

Treatment options were discussed. The dermatologist who performed the biopsy had completed a fellowship in Mohs micrographic surgery and recommended excision of the tumor using the Mohs micrographic surgical technique with microscopic evaluation of the margins during the removal of the cancer. I was on an anticoagulant and preferred to avoid a surgical scar. In addition, based on my professional experience when I had successfully treated patients who had a superficial basal cell carcinoma with topical 5% imiquimod cream, I requested that topical treatment using 5% imiquimod cream be used initially for the management of my basal cell carcinoma. If the cancer persisted or recurred, I agreed to return for excision of the neoplasm.

Daily topical treatment (performed each consecutive morning) using 5% imiquimod cream was started. The medication (12.5 mg of active drug in 250 mg of cream) was in a small packet intended for single-use. However, I would carefully place the open packet in a sealed zip-lock plastic bag after each use. During treatment, each packet of imiquimod (which contained 12.5 mg of drug in 250 mg of cream) would provide medication for approximately 7 to 10 days of therapy. The visual size of the drug applied each day to the face was like that of a sesame seed (3 mm to 4 mm long and 2 mm wide). Based on the total number of daily doses each packet provided, each daily treatment had between 1.25 mg per day (12.5 mg divided by 10 days) of drug in 25 mg per day (250 mg divided by 10 days) of cream (when the content of the packet of medication was completely used during a period of 10 days) to 1.79 mg per day (12.5 mg divided by seven days) of drug in 35.7 mg per day (250 mg divided by seven days) of cream (when the content of the packet of medication was completely used during a period of seven days).

**Figure 3:**
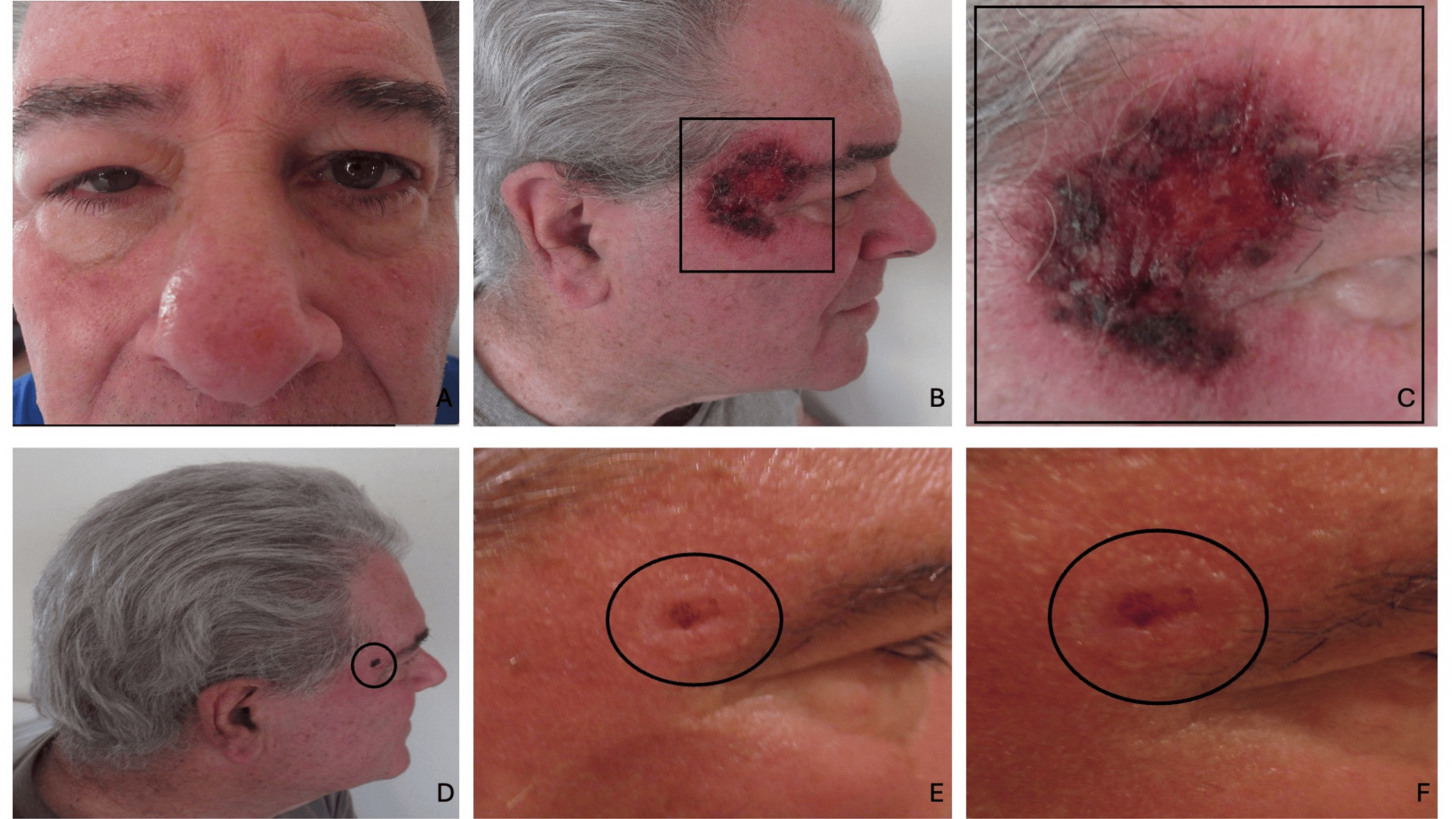
Cutaneous reaction to daily topical 5% imiquimod after nine treatments Frontal view (A), distal side view (B), and closer side view (C) of the right side of the face after nine topical applications of 5% imiquimod cream. There is edema of both the right upper and lower eyelids; the upper eyelid has more prominent swelling (A). Side views (B and C) of the face show the ulcer with not only surrounding eschar but also erythema peripheral to the eschar. Image C shows a closer view of the area in the square in image B. The imiquimod cream was stopped after nine applications. The treatment site was treated with a topical antibiotic; 2% mupirocin ointment was applied three times daily for 10 days. Six days after stopping the topical antibiotic, distant (D) and closer (E and F) views showed healing of the ulcer, resolution of the eschar, and no residual inflammation of the treatment site. However, the ulcerated tumor was still present (surrounded by the black oval). I consent to the publication of the images and my eyes not being blocked.

I applied the 5% imiquimod cream not only to the basal cell carcinoma but also to 2 mm to 3 mm of normal-appearing skin that surrounded the cancer. After nine days of treatment, I noticed marked edema of my right eyelids. There was an asymptomatic ulcer that had replaced the tumor nodule; eschar was present around the ulcer, and erythema surrounded the eschar (Figure 3).

I stopped the topical 5% imiquimod cream and began to apply 2% mupirocin ointment three times each day to the affected area of my face. After two weeks of treatment, the eyelid edema and treatment site reaction had resolved. However, residual neoplasm was still present (Figure 3).

**Figure 4:**
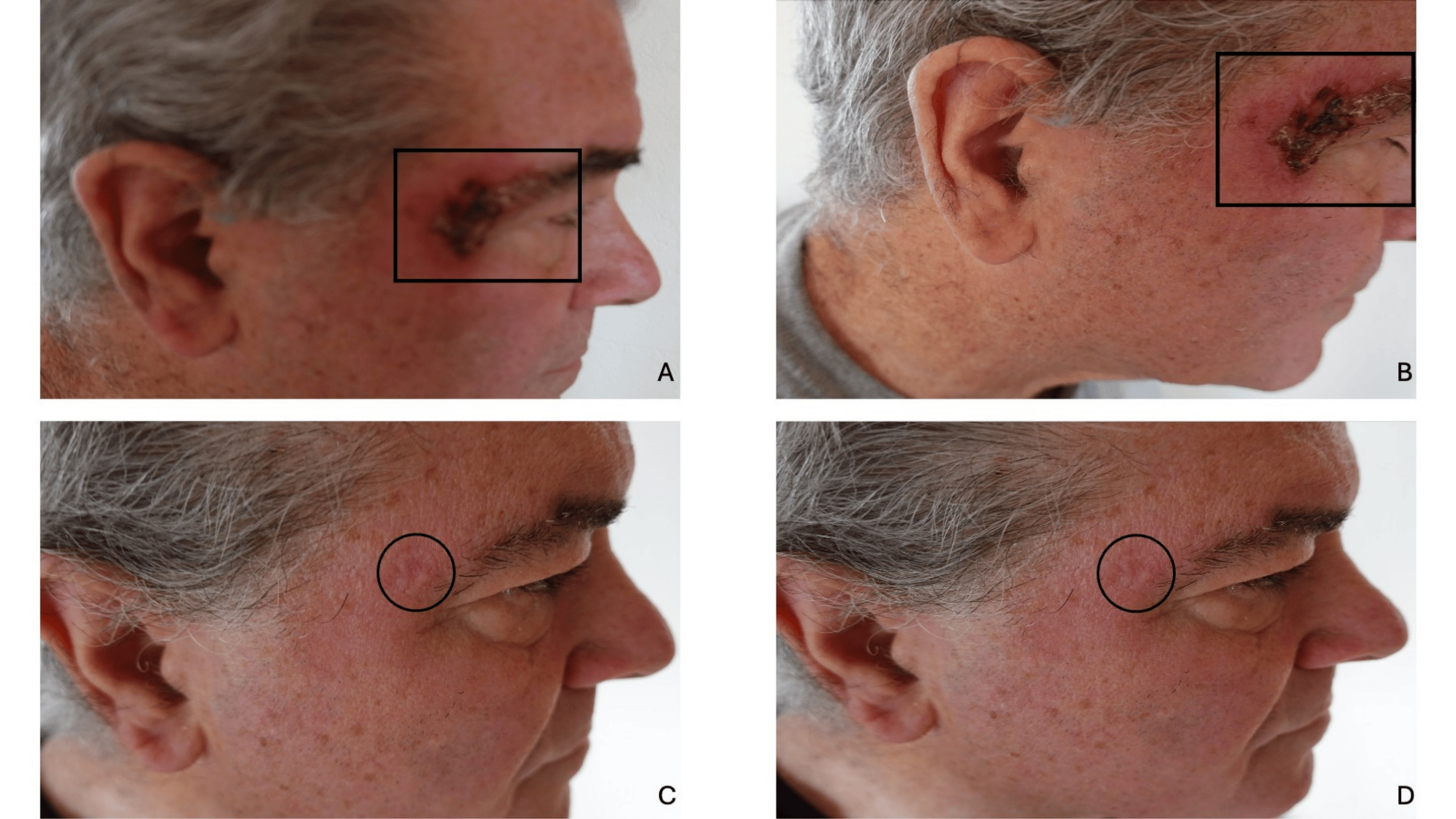
Imiquimod-induced cutaneous reaction at the treatment site of basal cell carcinoma Distant (A) and closer (B) views of the reaction to 18 daily applications of topical 5% imiquimod cream. The closer view (B) of the area within the black rectangle in the distant view (A) shows the ulcer, eschar, and surrounding erythema involving the right periorbital temple and the lateral right eyebrow. Distant (C) and closer (D) views acquired 18 days after the last topical treatment with imiquimod (which included five days of oral antibiotic and 10 days of topical antibiotic) show resolution of the eschar and erythema. However, ulceration is still noted in the center of the tumor (as demonstrated within the black oval). I consent to the publication of the images and my eyes not being blocked.

I waited for 10 weeks after stopping the immunotherapy to begin a second course of daily topical 5% imiquimod cream. After 18 days of treatment, the site of therapy was painful, and I stopped the topical application of 5% imiquimod cream. There was an ulcer, eschar, and surrounding confluent erythema on the right temple and the lateral right eyebrow (Figure 4).

I considered the possibility of a bacterial infection or superimposed impetiginization of the area. I started cephalexin 500 mg twice daily for five days and topical 2% mupirocin ointment three times daily for 10 days. After the oral and topical antibiotic treatment, the skin changes at the imiquimod treatment site had resolved; however, residual neoplasm was still present (Figure 4).

**Figure 5:**
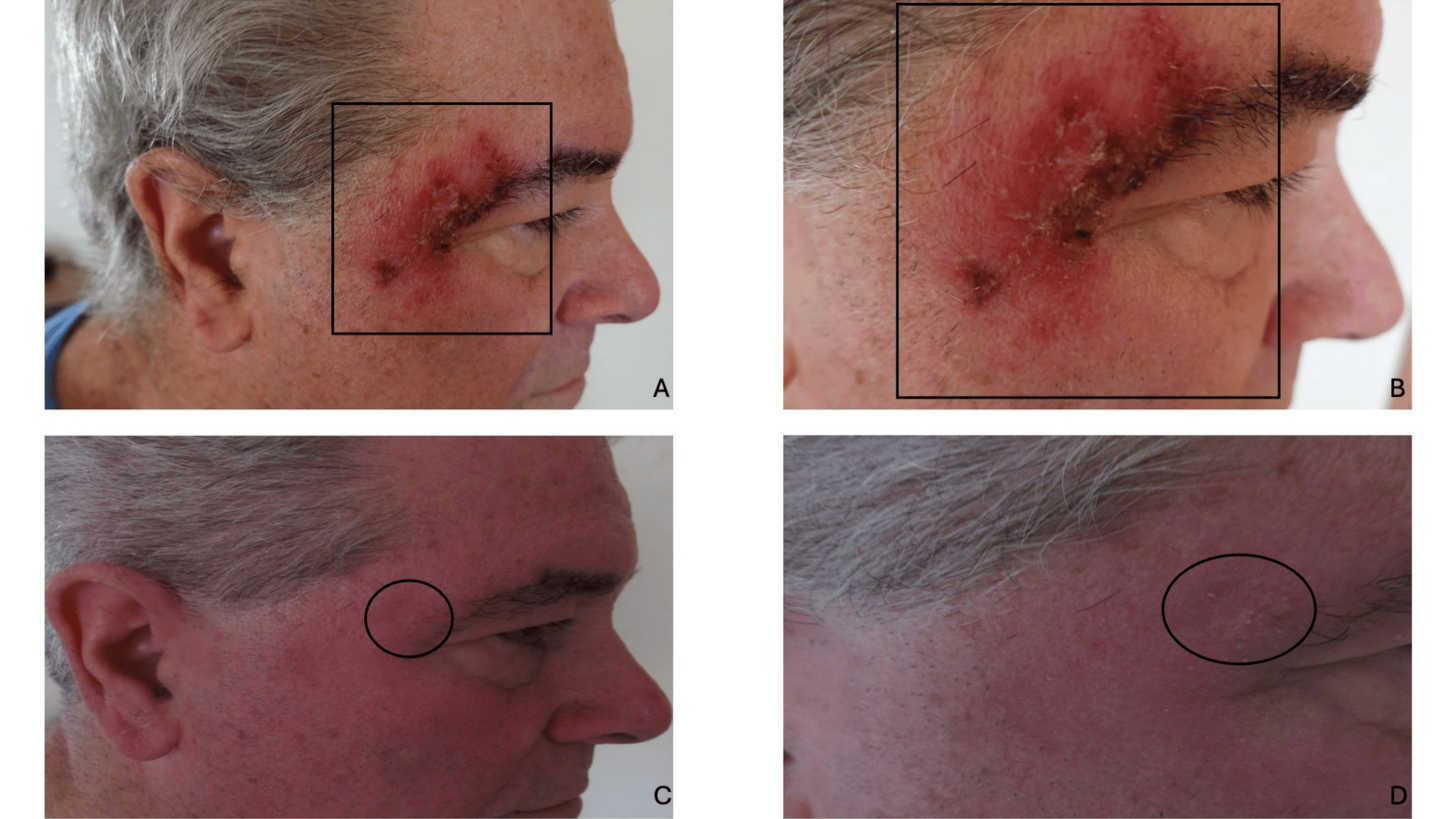
Cutaneous reaction resulting at the treatment site after the third course of topical imiquimod at the treatment site of a nodular basal cell carcinoma and tumor resolution Distant (A) and closer (B) views of the reaction to topical 5% imiquimod cream after 18 daily applications. The closer view (B) of the area in the black rectangle from the distant view (A) shows superficial erosions, eschar, and erythema on the right periorbital temple and the right lateral eyebrow. Distant (C) and closer (D) views of the right temple at the previous site of the nodular basal cell carcinoma; an atrophic patch (within the black oval) and no residual tumor were observed 25 days after the last topical treatment with 5% imiquimod cream. I consent to the publication of the images and my eyes not being blocked.

After stopping the immunotherapy, I waited for 18 days to begin a third course of daily topical 5% imiquimod cream. After another 18 days of treatment, I was again experiencing pain localized to the area where the 5% imiquimod cream had been applied. There were not only superficial erosions but also eschar and erythema on the right temple and the lateral right eyebrow (Figure 5). I stopped the topical application of 5% imiquimod cream.

Again, because the treatment site was painful, I took cephalexin 500 mg twice daily for five days and treated the area topically with 2% mupirocin ointment three times daily for 10 days. The skin changes at the imiquimod treatment site completely healed after the oral and topical antibiotic treatment. There was no residual neoplasm, and an atrophic patch was present at the previous site of the nodular basal cell carcinoma (Figure 5).

In summary, the standard treatment of a superficial basal cell carcinoma using topical 5% imiquimod cream is 30 applications (consisting of five consecutive daily applications during each period of seven days) over a period of six consecutive weeks. I had applied the medication 45 times, once on each consecutive day, during three sequential treatments which consisted of either nine days, 18 days, or 18 days. A follow-up seven months after the third course of topical 5% imiquimod treatment did not show any clinical recurrence of the tumor.

## Discussion

Basal cell carcinoma is a malignancy that almost exclusively affects the skin; however, infrequently, it may metastasize to visceral organs. There are several clinical morphologies that can be associated with basal cell carcinoma; they include predominantly the following types of tumors: fibroepithelial, morpheaform (which are also referred to as desmoplastic or sclerosing), nodular, pigmented, red dot, and superficial. Each of the clinical presentations of basal cell carcinoma has corresponding pathologic features; indeed, other pathologic types (such as infiltrative, micronodular, and basosquamous) and tumors of mixed histology (consisting of two or more pathologic subtypes) may occur [1-4].

There are various treatment options available to treat basal cell carcinoma. Management may be influenced by not only the location of the cancer, but also the tumor’s clinical presentation and pathologic subtype [1-4]. The gold standard of treatment for most basal cell carcinoma is surgical excision [1,6,7,11].

Localized tumor or nonaggressive pathology subtypes of basal cell carcinoma on the trunk or extremities may be treated with either monotherapy or combination treatment using topical agents, locally destructive techniques, or other modalities such as photodynamic therapy or laser [5,12,13]. In contrast, tumors with aggressive pathology subtypes or at sites more prone to clinical recurrence are typically excised with or without microscopic examination of the margins during surgery [4-7]. Advanced basal cell carcinoma or metastatic basal cell carcinoma may need to be treated with systemic hedgehog inhibitors or immunotherapeutic agents [1,3,4,14].

Basal cell carcinoma on the face is often treated with excision using Mohs micrographic surgery [3]. The margins are evaluated during the removal of the tumor [4]. Additional tissue is excised specifically at the locations where residual cancer is observed until the tumor has been completely excised [6]. The cure rates for basal cell carcinomas treated using Mohs surgery are approximately 97.5% (after five years) and 95.6% (after 10 years) for primary tumors and about 97.6% (after five years) and 96.1% (after 10 years) for recurrent neoplasms [1,4].

Imiquimod, an imidazoquinoline, has antiviral and antitumor activity. It is postulated that the drug modifies the immune response and results in the induction of apoptosis in keratinocytes of basal cell carcinoma; however, the definitive mechanism of action of its antineoplastic activity remains to be established. Erythema, erosions, eschar formation (presenting as crusting and scabbing), and ulcers are frequent cutaneous adverse events associated with the topical use of 5% imiquimod cream for the treatment of basal cell carcinoma [8-10].

In the United States of America, topical 5% imiquimod cream is approved by the Food and Drug Administration for the treatment of extragenital and perianal warts (once every other day, three times per week, and for a maximum of 16 weeks) and actinic keratoses (that are non-hyperkeratotic and non-hypertrophic) on the face and scalp of immunocompetent adults (once on two days per week, separated by two days, and for a full 16-week cycle). Imiquimod 5% cream has also been approved for the topical treatment of primary superficial basal cell carcinomas on the neck, trunk, and extremities of immunocompetent adults when used once daily, five times per week for six consecutive weeks [8].

The topical application of 5% imiquimod cream has been evaluated for the management of not only superficial basal cell carcinomas. It has also been evaluated for periocular and nonperiocular nodular basal cell carcinomas. In addition, the effectiveness of topical 5% imiquimod cream has been compared to topical 5% 5-fluorouracil cream and photodynamic therapy in patients with superficial basal cell carcinomas [11-19].

In a prospective, noninferiority, randomized controlled multicentered trial that included 601 patients with superficial basal cell carcinoma, topical 5% imiquimod cream was superior to both topical 5% 5-fluorouracil cream and methyl aminolevulinate photodynamic therapy. The probability of tumor-free survival was 80.5% for imiquimod, 70.0% for 5-fluorouracil, and 62.7% for photodynamic therapy. The investigators concluded that, in terms of efficacy, 5% imiquimod cream should be considered as the first choice for a noninvasive treatment for most primary superficial basal cell carcinomas [12].

Imiquimod 5% cream has demonstrated 82% histologic clearance of superficial basal cell carcinomas with a five-times weekly application. Nodular basal cell carcinomas, treated five times weekly, showed lower clearance rates ranging from 42% to 100%. The clearance rates for infiltrative basal cell carcinomas, treated with daily applications five times weekly, also showed slower clearance rates than superficial basal cell carcinomas and ranged from 56% to 63% [8].

Studies comparing low-frequency dosing (either two or three days per week) with and without occlusion were conducted using topically applied 5% imiquimod cream for the treatment of either superficial (93 patients) or nodular (90 patients) basal cell carcinomas. The patients were evaluated six weeks following a six-week treatment period. The highest complete response rate histologically was seen in the three days per week with occlusion for both superficial (87%) and nodular (65%) basal cell carcinomas. The investigators noted that the 87% response rate that they observed after applying imiquimod three days per week with occlusion was similar to the response rate without occlusion observed in either patients who received 12 or six weeks of daily treatment or in individuals who were treated 5 days per week for 12 weeks [18].

A systematic review of seven studies, which included 152 periocular superficial or nodular basal cell carcinomas, assessing topical 5% imiquimod cream demonstrated a pooled clinical-plus-histological clearance across case series of 82%. Local adverse effects of the treatment were observed in 85 of 122 patients (more than 70% of the participants). The adverse reactions included erythema, crusting, or conjunctivitis. The researchers concluded that, when surgery is contraindicated, or cosmesis is a paramount concern, topical 5% imiquimod cream is a useful, tissue-sparing option for selected superficial and nodular periocular basal cell carcinomas [19].

Another group of investigators did a comparison of dosing regimens to evaluate the efficacy of topical 5% imiquimod cream for the treatment of nodular basal cell carcinomas. Imiquimod was applied once daily for either three, five, or seven days for either six or twelve weeks. The highest clearance rates of 71% (25 of 35 patients) and 76% (16 of 21 patients) were observed with dosing of topical 5% imiquimod cream once daily for seven days per week after six and 12 weeks, respectively. The researchers concluded that for the treatment of nodular basal cell carcinoma, 5% imiquimod cream applied once daily for seven days per week for either 12 or 6 weeks, was not only well tolerated but also an effective treatment [15].

A non-randomized trial demonstrated an efficacy of 5% imiquimod cream in the treatment of periocular nodular basal cell carcinomas. Treatment consisted of 5% imiquimod cream applied topically five days per week for 8 to 16 weeks; 19 of 24 patients remained until the end of treatment. Evaluation was done at three months and 39.5 months after completion of therapy. The histologic clearance rate was 89.5% after three months and was 84.2% after three years. The investigators noted that the histologic clearance rate was 100% (eight of eight lesions) when the tumor was less than 10 mm in diameter and was only 81.8% (9 of 11 tumors) when the cancer was more than 10 mm in diameter [16].

Multiple investigators were involved in a single-blind, noninferiority randomized controlled surgery versus imiquimod for nodular and superficial basal cell carcinoma (SINS) trial. Patients were randomized to either excisional surgery (with a 4 mm margin of surrounding normal-appearing skin) or 5% imiquimod cream (applied topically once daily for either six weeks for a superficial basal cell carcinoma or 12 weeks for a nodular basal cell carcinoma). The five-year success rate without recurrence was 97.7% (173 of 177 individuals) for surgery and 82.5% (170 of 206 individuals) for imiquimod [13,17].

In the SINS trial, the cure rate using 5% imiquimod cream was inferior to surgery for the treatment of basal cell carcinoma; yet, the investigators observed a sustained benefit for the lesions that responded early to topical imiquimod [17]. However, in several studies, in spite of the lower rates of cure using imiquimod, patients had a preference for topical therapy with imiquimod to treat their cancer in comparison to excision of the tumor. The reasons for this preference were a presumed better cosmetic outcome for the noninvasive imiquimod treatment and concerns about possible side effects that might result from surgical excision [17,20]. 

For most patients whom I diagnosed with a basal cell carcinoma, excision of the tumor was recommended. However, I also treated several patients with a superficial basal cell carcinoma on the extremity with topical 5% imiquimod cream. Most patients with a superficial basal cell carcinoma on their extremities who were treated with topical 5% imiquimod cream had an excellent response to treatment that was characterized by complete tumor clearing. 

In addition, I recall an older woman with a large superficial and nodular basal cell carcinoma on the sidewall of her nose, in whom surgery was contraindicated, who had an excellent clinical response with complete resolution of her tumor with 5% imiquimod cream. Like me, she also had a severe inflammatory response to the topical medication. Her therapy had to be discontinued prior to six weeks of treatment, and she was treated with oral antibiotics for a clinically suspected bacterial infection of the treatment site.

A comparison of topical application of 5% imiquimod cream and Mohs micrographic surgery excision for the management of basal cell carcinoma is summarized in Table 1 [1-4,6,8-11,13,14,17-19]. Several factors, including tumor subtype and location, should be considered when determining the most appropriate approach to therapy for a patient with basal cell carcinoma. Both approaches to the treatment of basal cell carcinoma have advantages and disadvantages.

**Table 1:** Comparison of topical 5% imiquimod cream and Mohs micrographic surgery for the treatment of basal cell carcinoma

Characteristic	Topical 5% imiquimod cream	Mohs micrographic surgery	References
Tumor subtype	Predominantly superficial, occasionally nodular	All (including superficial, nodular, and infiltrative)	[1-4,6,8-11,13,14,17-19]
Location of tumor	Extremities and trunk (areas at low risk for post-treatment persistence)	Face, nose, and periocular (areas at high risk for post-treatment persistence)	[1-4,6,8-11,13,14,17-19]
Rate of success	Superficial subtype: 82%, nodular subtype: 42-100%, infiltrative subtype: 56-63%	97.5%	[1-4,6,8-11,13,14,17-19]
Recurrence	Higher rate of tumor persistence	Very low rate of tumor persistence	[1-4,6,8-11,13,14,17-19]
Invasive treatment	No	Yes	[1-4,6,8-11,13,14,17-19]
Procedure	Daily topical application of a cream	Surgical excisions until clear margins are obtained (and local anesthesia)	[1-4,6,8-11,13,14,17-19]
Who performs the procedure?	Patient	Physician	[1-4,6,8-11,13,14,17-19]
Duration of treatment	Superficial subtype: six weeks, nodular subtype: up to 12 weeks	Usually, one day (and may take several hours). repair of the wound is often the same day or at a subsequent visit	[1-4,6,8-11,13,14,17-19]
Surgical scar	No	Yes	[1-4,6,8-11,13,14,17-19]
Post-treatment cosmetic result	Typically excellent, skin alteration may occur	Depends on the size of the wound after removal of the tumor	[1-4,6,8-11,13,14,17-19]
Potential adverse effects	Crusting, erosion, erythema, eschar, infection, inflammation, pain, and ulcer	Erythema, graft necrosis, infection, scar, and wound dehiscence	[1-4,6,8-11,13,14,17-19]

The advantages of topical 5% imiquimod cream for basal cell carcinoma treatment are that it is a noninvasive modality that can be performed by the patient. In addition, it is a potential method of therapy for patients for whom surgery is contraindicated. There are several disadvantages of treating with the topical 5% imiquimod cream. Some of these include the lower rate of success, the long duration of treatment, and the adverse cutaneous reactions that may occur at the site of treatment [8-11,13,14,17-19].

The advantages of Mohs micrographic surgery are the high rate of tumor clearance. In addition, surgery has a low rate of carcinoma persistence after cancer-free margins have been obtained during the procedure. In contrast to topical 5% imiquimod cream, in which the patient is responsible for ensuring continued compliance with treatment, the excision is performed by a physician who conducts the procedure and evaluates the tissue to determine that the tumor has been removed [1-4,6].

Another advantage of surgical excision of the cancer is the shorter duration of treatment. In some cases, the removal of the tumor using Mohs micrographic surgery may require multiple excisions to ensure histologic clearance of the neoplasm. This is usually accomplished within the same day. However, in some circumstances, when the closure of the surgical wound is more complex, another physician may be required to perform one or more advanced surgical procedures [1-4,6].

Disadvantages of Mohs micrographic surgery are that it is an invasive procedure that requires cutting into the skin and the removal of the tumor-containing skin. The dimensions of the cancer and the extent of tumor invasion into the dermis or subcutaneous tissue will determine the size of the surgical wound. The larger the wound, the more likely there may be an impact on the cosmetic result. Another disadvantage is potential postoperative surgical complications [1-4,6].

## Conclusions

There are several potential modalities that can be used for the management of basal cell carcinoma. The choice of therapy is influenced by the biological behavior of the neoplasm. The histologic subtype and location of the tumor are factors that contribute to the aggressiveness of the cancer. I, a 65-year-old man, developed a nodular basal cell carcinoma on my right periocular temple. I elected a nonsurgical treatment of my neoplasm. I daily applied 5% imiquimod cream topically to the tumor and adjacent skin for 45 days; therapy was temporarily discontinued twice to manage the local cutaneous adverse side effects associated with the medication. Complete clinical clearance of the tumor was achieved with a focal atrophic patch at the previous tumor site. Mohs micrographic surgery is the treatment of choice for most facial basal cell carcinomas and tumors of infiltrative histologic subtype; it is associated with an excellent cure rate of about 97.5% for primary basal cell carcinomas and a very low rate of tumor persistence. Although Mohs micrographic surgery is considered to be an invasive procedure and can be associated with postoperative complications, scar, and possibly inferior cosmetic results, it is usually the preferred treatment for basal cell carcinoma subtypes that are associated with a higher rate of clinical recurrence. Daily topical application of 5% imiquimod cream for six weeks for basal cell carcinoma on the neck, trunk, or extremities is an approved treatment modality for superficial basal cell carcinomas. In addition, although the cure rate is lower, this therapy has also been used for nodular basal cell carcinomas, including facial tumors such as those that are periocular. The advantages of daily topical treatment with 5% imiquimod cream are that it is a noninvasive therapeutic approach and may result in a cosmetically superior result in contrast to surgical excision. The disadvantages of topical 5% imiquimod cream as compared to an excision using the Mohs micrographic surgical technique are the lower cure rate, the extensive duration of treatment (such as five daily applications each week for six weeks to treat a superficial basal cell carcinoma), and the development of adverse cutaneous reactions localized not only to the tumor-containing epithelium, but also the skin surrounding the neoplasm. In summary, there are noninvasive and invasive modalities for the management of basal cell carcinomas; several factors influence the decision of the physician and patient in determining the treatment of a basal cell carcinoma. Although surgical excision may be the treatment of choice for most basal cell carcinomas, in some circumstances, noninvasive approaches, such as topical 5% imiquimod cream, may be appropriate.
